# Multimodal Classification of Alzheimer’s Disease Using Longitudinal Data Analysis and Hypergraph Regularized Multi-Task Feature Selection

**DOI:** 10.3390/bioengineering12040388

**Published:** 2025-04-05

**Authors:** Shuaiqun Wang, Huan Zhang, Wei Kong

**Affiliations:** College of Information Engineering, Shanghai Maritime University, 1550 Haigang Ave., Shanghai 201306, China; 202230310047@stu.shmtu.edu.cn (H.Z.); weikong@shmtu.edu.cn (W.K.)

**Keywords:** multi-task learning, longitudinal data, hypergraph learning, multimodal classification, Alzheimer’s disease

## Abstract

Alzheimer’s disease, an irreversible neurodegenerative disorder, manifests through the progressive deterioration of memory and cognitive functions. While magnetic resonance imaging has become an indispensable neuroimaging modality for Alzheimer’s disease diagnosis and monitoring, current diagnostic paradigms predominantly rely on single-time-point data analysis, neglecting the inherent longitudinal nature of neuroimaging applications. Therefore, in this paper, we propose a multi-task feature selection algorithm for Alzheimer’s disease classification based on longitudinal imaging and hypergraphs (THM2TFS). Our methodology establishes a multi-task learning framework where feature selection at each temporal interval is treated as an individual task within each imaging modality. To address temporal dependencies, we implement group sparse regularization with two critical components: (1) a hypergraph-induced regularization term that captures high-order structural relationships among subjects through hypergraph Laplacian modeling, and (2) a fused sparse Laplacian regularization term that encodes progressive pathological changes in brain regions across time points. The selected features are subsequently integrated via multi-kernel support vector machines for final classification. We used functional magnetic resonance imaging and structural functional magnetic resonance imaging data from Alzheimer’s Disease Neuroimaging Initiative at four different time points (baseline (T1), 6th month (T2), 12th month (T3), and 24th month (T4)) to evaluate our method. The experimental results show that the accuracy rates of 96.75%, 93.45, and 83.78 for the three groups of classification tasks (AD vs. NC, MCI vs. NC and AD vs. MCI) are obtained, respectively, which indicates that the proposed method can not only capture the relevant information between longitudinal image data well, but also the classification accuracy of Alzheimer’s disease is improved, and it helps to identify the biomarkers associated with Alzheimer’s disease.

## 1. Introduction

Alzheimer’s disease (AD) is a chronic neurodegenerative disease that is the leading cause of dementia, leading to language problems, disorientation, mood swings, physical dysfunction, and ultimately death [1]. Many studies have shown [2,3,4] that the number of AD patients and the costs of AD-related treatment and care are rapidly increasing. AD has a serious effect on patient safety and socioeconomic well-being. Although some treatments can temporarily improve symptoms of AD, there is no treatment to date that can stop or reverse its progression. Therefore, the early diagnosis of AD and its precursor symptom, mild cognitive impairment (MCI), is important for the timely treatment of this disease.

Over the past few decades, neuroimaging techniques have proven to be a powerful tool for studying the differences in the characteristics of neurodegenerative progression between AD and normal controls. With the rapid development of artificial intelligence, various improved machine learning methods have been widely used in the diagnosis of AD, making great contributions to the use of neuroimaging data to assist doctors in AD diagnosis. Existing studies using the combination of machine learning and brain image data to distinguish between healthy elderly people and AD patients have achieved good results. For example, Uysal et al. [5] created a dataset based on age, gender, and left and right hippocampal volume values, and then they classified hippocampal volume information using machine learning techniques. The experiment proved that magnetic resonance imaging (MRI) images could be used to distinguish AD, MCI, and normal controls (NCs). Fan et al. [6] proposed a U-net model for AD classification using MRI images. Jia et al. [7] designed a two-modal model of fusion functional magnetic resonance imaging (fMRI) and structural functional magnetic resonance imaging (sMRI). The method processes mReHo and structural MRI data, respectively, through two simplified 3D Convolutional Neural Network (3D-CNN) architectures, uses nuclear canonical correlation analysis (KCCA) for multimodal feature fusion, and finally realizes disease classification using a support vector machine (SVM) classifier.

Medical image data are the key in AD-related research, which is often high and noisy with many redundant or irrelevant features. If these features are not processed well, the model performance will be poor. Feature selection can solve the problem of high and noisy medical image data by reducing the feature dimension and removing irrelevant features. In recent years, multimodal feature selection methods have made significant progress in AD computer-aided diagnosis, and many studies have verified that data from different modes, providing necessary complementary information to improve model performance. For example, Shao et al. [8] proposed a multi-task feature selection method based on a hypergraph for AD/MCI classification and introduced regularization terms based on a hypergraph into the standard multi-task feature selection to model the higher-order structural relationship between subjects and to obtain better classification performance. Shi et al. [9] proposed a novel multimodal feature selection method called Adaptive Similarity Multimodal feature selection (ASMFS), which performs adaptive similarity learning and feature selection simultaneously. Ban et al. [10] proposed a hypergraph *p*-Laplacian regularization multi-task feature selection algorithm for AD classification. The algorithm introduced hypergraphs to retain higher-order structure information of similar data and Frobenius norm regularization terms to improve the model’s anti-noise performance, and the results showed that the algorithm achieved good classification results.

Although the above studies have achieved good results, the existing methods only use baseline data for prediction and ignore the longitudinal changes in the brain. Since hypergraphs are a common method to capture information about the higher-order structure of data, they can be used to model complex relationships between individuals through the connection between hyperedges and vertices [11]. They are also widely used in computer vision, bioinformatics and medical image analysis [12,13]. Therefore, this study proposes a multi-task feature selection method called the longitudinal imaging and hypergraph-based multi-task feature selection algorithm (abbreviated as THM2TFS). The feature selection of each time point in a specific modality is treated as an independent task, and a group sparse regularization algorithm is used to jointly extract common features across multiple time points. Additionally, we have developed a Fused Lasso regularization term that aims to incorporate temporal smoothness constraints between adjacent time points’ feature weights, further reflecting the longitudinal progressive changes in brain regions. In addition, by using the Laplacian matrix of hypergraphs and embedding hyperedges with K-nearest neighbors (KNN), each feature is treated as a vertex to preserve high-order structural relationships among samples of the same modality. Finally, we utilized a multicore support vector machine to fuse multimodal features and perform the final classification. To validate the effectiveness of our proposed method, we conducted experiments on the Alzheimer’s Disease Neuroimaging Initiative (ADNI) dataset. The results showed that our method is more effective than the initial approach.

## 2. Method

**Figure 1:**
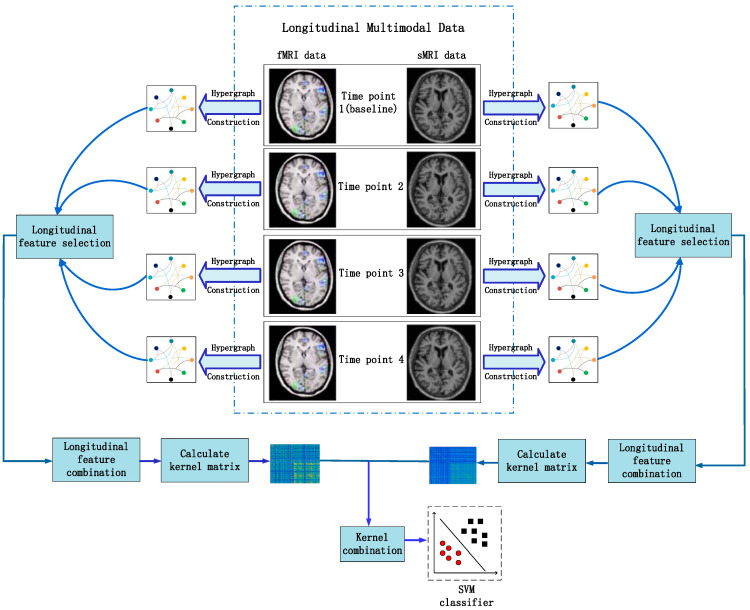
Classification framework of the proposed method.

The framework of the method is illustrated in Figure 1. It can be observed from Figure 1 that the classification framework can be roughly divided into four parts, namely, data preprocessing, hypergraph construction, multimodal longitudinal feature selection, and classification. Specifically, the longitudinal image data of AD patients, MCI patients, and NC subjects with both fMRI images and sMRI images were first selected in the ADNI database as study objects. Secondly, the fMRI images and sMRI images of the selected subjects were preprocessed, and 90 regions of interest (ROIs) of the fMRI images and sMRI images were extracted, respectively, through the AAL brain atlas as the pre-selected features of the brain images. Then, based on the extracted features, a hypergraph was built for the four time-point data of each mode to capture the higher-order structure information of the data. Then, the most discriminative common features were selected from the preselected features through a multi-task feature selection algorithm based on longitudinal imaging and hypergraph. Finally, the features of the two modes were fused and classified using multi-kernel SVM. Below, this chapter will describe the details of each step.

### 2.1. Data Collection and Preprocessing

#### 2.1.1. Data Collection

Regarding database for performance evaluation, this study was led by the National Institutes of Health (NIH), joined with numerous agencies who funded support of the ADNI database (http://ADNI.loni.usc.edu/ (accessed on 15 March 2022)). As a database project with a public nature, it adopts a longitudinal multi-center observation method to carry out research. The database integrates data from more than 8000 volunteers from more than 100 clinical facilities across North America, covering a range of cognitive states. These include older adults with normal cognitive function, those diagnosed with MCI, and those already diagnosed with AD. The information in the database is very rich and contains many aspects. In terms of image data, there are multimodal image data such as structural magnetic resonance imaging (sMRI) and positron emission tomography (PET). Biological data cover cerebrospinal fluid, blood, brain tissue, and other biological sample resources; in addition, multi-dimensional data such as clinical evaluation records, cognitive function test results, and genetic testing information were integrated [14,15]. Since its establishment, the database has provided strong data support for the research and development of early diagnosis technology and clinical treatment optimization of Alzheimer’s disease and its related diseases through an open and shared mechanism and has strongly promoted research progress in related fields.

In this study, we collected neuroimaging data from 512 participants of the ADNI, including fMRI and sMRI data at four different time points: baseline (T1), 6th month (T2), 12th month (T3), and 24th month (T4). The participants consisted of 136 Alzheimer’s disease patients, 200 individuals with MCI, and 176 normal controls. For longitudinal data loss and incomplete data, neuroimages of AD patients can be generated automatically to solve the missing or incomplete data in this study [16]. Here, we used generative adversarial networks (Gans) to generate meaningful data [17]. Table 1 presents the demographic information of the participants.

#### 2.1.2. Data Preprocessing

From the ADNI database, we retrieved fMRI and sMRI data samples with time distributions at month 0 (T1), month 6 (T2), month 12 (T3), and month 24 (T4). After selection, we eliminated brain imaging samples with indiscernible structures and samples with image distortion, leaving a total of 512 remaining samples. After selection, we eliminated brain imaging samples with difficult-to-discern structures and distorted image samples, leaving a total of 512 remaining samples. Subsequently, the four brain images of each sample were subjected to head motion correction, segmentation, registration, and feature extraction using the SPM12 program in MATLAB R2018b. After 116 ROIs were extracted using the automated anatomical labeling (AAL) template, 26 cerebellar structures were eliminated, and 90 ROIs were retained as the final features of the brain image [18]. After statistical evaluation, there were no statistically significant differences in age and gender between the two groups.

### 2.2. Hypergraph Construction

In many applications, researchers use hypergraphs to represent higher-order relationships. Hypergraphs can connect two or more vertices together through hyperedges, allowing for the description of higher-order relationships between entities. A hypergraph is a high-dimensional graphical representation of data that compensates for information loss when using ordinary graphs. Therefore, hypergraphs have significant advantages in modeling the correlation of real data, which may be much more complex than pairwise relationships. Currently, graph learning has been widely applied in many practical applications.

In order to express the higher-order relationship between subjects, we constructed hypergraphs at four time points. In this paper, Euclidean distance was used to measure the correlation strength between nodes [19]. Then, the KNN method was used to construct a hypergraph, with each node regarded as the central node, and k nodes connected to each central node were calculated through KNN algorithm [20], so as to construct hypergraph. Therefore, the complete hypergraph that we constructed is the set of N (number of subjects) vertices and N hyperedges, all of which weigh 1.

**Figure 2:**
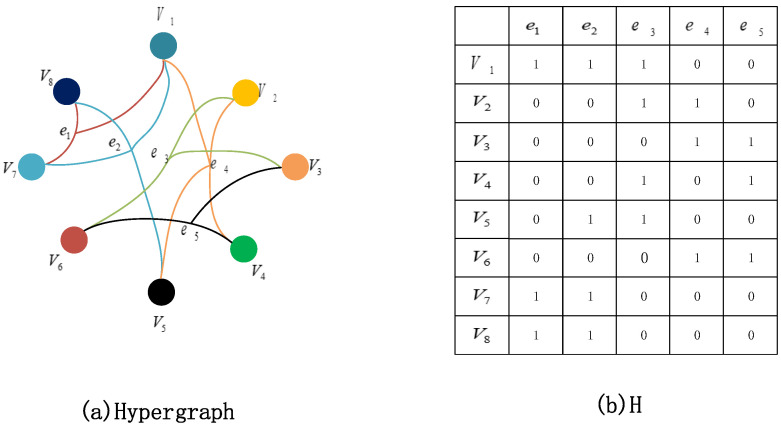
Hypergraph model and corresponding incidence matrix.

As shown in Figure 2, each hyperedge contains multiple nodes, and the nodes contained between hyperedges can be duplicated. The connected vertices are linked by edges of the same color. In particular, when a hyperedge contains only two vertices, it degenerates into a regular edge. Therefore, simple graphs are a special type of hypergraph.

In mathematics, a hypergraph is represented by G (V, E, W), where V is the set of vertices, E is the set of hyperedges, and W is the set of weights assigned to the hyperedges. Each hyperedge $e_{i}=(i=1,2,\cdots N_{e})$ is assigned a weight $w(e_{i})$. A hypergraph G is usually represented by an incidence matrix H, which is defined as follows:

(1) $$H(v,e)=\left\{\begin{array}{c} 1,\;\;if\;v\in e\\ 0,\;\;if\;v\notin e \end{array}\right.$$

When vertex v is present in hyperedge e, the value of incidence matrix H is 1; otherwise, it is 0. The geometric interpretation of the hypergraph is shown in Figure 2a, and the association matrix H is shown in Figure 2b.

In a hypergraph, the sum of the weights of hyperedges to which vertex v belongs is referred to as the degree of vertex v, which is denoted as follows:

(2) $$d=\sum_{e\in E}w(e)H(v,e)$$

The number of vertices contained in a hyperedge e is referred to as the degree of the hyperedge e, denoted as follows:

(3) $$\delta (e)=\sum_{v\epsilon V}H(v,e)$$

In addition, we use the diagonal matrices $D_{v}$ and $\;D_{e}$ to represent the degree matrices of vertices and hyperedges, respectively, defined as follows:

(4) $$D_{v}=diag\left(d\left(v_{1}\right),d\left(v_{2}\right),\cdots d\left(v_{N}\right)\right)$$

(5) $$D_{e}=diag\left(\delta \left(e_{1}\right),\delta \left(e_{2}\right),\cdots \delta \left(e_{N}\right)\right)$$

Let W represent the diagonal matrix of weights for each hyperedge, defined as

(6) $$W=diag\left(w\left(e_{1}\right),w\left(e_{2}\right),\cdots w\left(e_{N}\right)\right)$$

In this paper, we use the method proposed in reference [21] to calculate the hypergraph Laplacian matrix:

(7) $$L^{h}=I-\Theta$$

(8) $$\Theta =D_{v}^{-1/2}HWD_{e}^{-1}H^{T}D_{v}^{-1/2}$$

where $L^{h}$ is the Laplacian matrix of the hypergraph, and I is the identity matrix.

### 2.3. Longitudinal Feature Selection

#### 2.3.1. Multi-Task Feature Selection

Multi-task learning [22] is a method that improves the performance of a specific model by simultaneously learning related but distinct tasks and leveraging the shared information between them. In longitudinal data research, the multi-task learning model can combine image data at different time points to find potential associations between different time points and improve the generalization ability of the model.

Suppose we have T learning tasks (i.e., T time points), where the t-th task consists of N data samples, and its data matrix can be represented as $X_{t}=\left[x_{1t};x_{2t};\cdots ;x_{Nt}\right]\in R^{N\times d}$. The corresponding class label is the following: $Y=[y_{1}{;y}_{2};\cdots y_{N}]\in R^{N}$. The variable $x_{it}\in R^{d}$ represents the i-th sample at time point t, and y∈{−1,+1} represents the class label of the corresponding sample. Let $w_{t}\in R^{d}$ be a column vector representing the parameters of the t-th task’s linear classification function. The objective function of the multi-task feature selection model is as follows:

(9) $$\underset{W}{\min}\frac{1}{2}\sum_{t=1}^{T}{\left\|Y-X_{t}w_{t}\right\|}_{2}^{2}+\beta {\left\|W\right\|}_{2,1}$$

where $W=[w_{1},w_{2},\cdots w_{T}]\in R^{d\times T}$ is a weight matrix composed of coefficient vectors at all time points. The parameter β is a regularization coefficient greater than zero, which balances the relative contributions of the group linear fitting loss term and group sparse regularization term to the objective function. In the equation, the first term is the empirical risk function, where a squared loss term is used to measure the difference between Y and $X_{t}w_{t}$. The second term is the group sparsity regularization, where ${\left\|W\right\|}_{2,1}$ represents the $l_{2,1}$ norm of matrix W. In longitudinal data research, $l_{2,1}$ norm is often used to find the most discriminative feature between different tasks, which is defined as the following:

(10) $${\left\|W\right\|}_{2,1}=\sum_{i=1}^{d}{\left\|w^{i}\right\|}_{2}$$

The $l_{1}$ norm ensures the sparsity of the model. The $l_{2}$ norm guarantees a pattern of similarity between all time points to ensure that the same brain region features are selected at the same time.

#### 2.3.2. Fused Lasso Regularization Model

Fused Lasso is a commonly used regularization technique in signal processing and bioinformatics, aimed at facilitating feature selection and estimation in signals with smooth variations over time or space. This technique can be seen as an extension of Lasso, as it not only selects predictive variables highly correlated with the response variable but also encourages neighboring predictive variables to have similar coefficient values, resulting in smooth estimates [23]. The Fused Lasso regularization term is defined as follows:

(11) $${\left\|W\right\|}_{F1}=\sum_{t=1}^{T_{t}-1}{\left\|w_{t+1}-w_{t}\right\|}_{1}$$

This regularization term tends to limit the difference between two consecutive normalized weight vectors at adjacent time points, promoting smoothness in the selection of neighboring features. The use of the $l_{1}$ norm in the fusion smoothing term encourages sparsity in the weight vector, resulting in a significant number of zero components. In other words, due to the use of fusion smooth regularization, many components in adjacent weight vectors will be identical. In this study, we will extract the feature subset with non-zero weight for the subsequent classification model training.

#### 2.3.3. Multi-Task Feature Selection Based on Longitudinal Imaging and Hypergraph

This article incorporates hypergraph regularization and Fused Lasso regularization terms into the objective formula for multi-task feature selection. Based on the Laplacian matrix of hypergraphs, we define a regularization term for hypergraphs.

(12) $$\Omega =w^{T}X^{T}L^{h}Xw$$

Therefore, the objective function of our TH2MTFS method is defined as follows:

(13) $$\underset{W}{\min}\frac{1}{2}\sum_{m=1}^{M}{\sum_{t=1}^{T_{t}}\left\|Y-X_{\mathrm{mt}}w_{\mathrm{mt}}\right\|}_{2}^{2}+\beta {\left\|W\right\|}_{2,1}+\lambda \sum_{m=1}^{M}\sum_{t=1}^{T_{t}}(X_{\mathrm{mt}}w_{\mathrm{mt}}{)}^{T}L_{\mathrm{mt}}^{h}\left(X_{\mathrm{mt}}w_{\mathrm{mt}}\right)+\mu {\sum_{m=1}^{M}\left\|\sum_{t=1}^{T_{t}-1}\left(w_{t+1}-w_{t}\right)\right\|}_{1}$$

where $W=[w_{1},w_{2},w_{3},w_{4}]$, M=2, and $T_{t}$ = 4. $L_{mt}^{h}$ is the Laplacian matrix of the hypergraph at time point t in mode m. The first term in the equation represents the empirical risk error on the training set, the second term is the $l_{2,1}$ norm, the third term is a hypergraph regularization term, and, finally, there is a Fused Lasso regularization term. In our model, we not only consider learning from data at a single time point but also incorporate data from multiple time points, which can be used for longitudinal analysis methods to provide a better diagnosis of AD. Additionally, a hypergraph regularization term was added to preserve the higher-order structural information in each temporal image.

#### 2.3.4. Algorithm Optimization

The objective formula of this article combines group sparsity, a hypergraph regularization term, and Fused Lasso regularization term, which cannot be solved using existing sparse learning models. In this paper, we employed the APG algorithm [24,25] to solve the objective formula. First, the objective function was divided into its smooth part:

(14) $$h\left(W\right)=\underset{W}{min}\frac{1}{2}{\sum_{m=1}^{M}\sum_{t=1}^{T_{t}}\left\|Y-X_{mt}w_{mt}\right\|}_{2}^{2}+\lambda \sum_{m=1}^{M}\sum_{t=1}^{T_{t}}(X_{mt}w_{mt}{)}^{T}L_{mt}^{h}\left(X_{mt}w_{mt}\right)$$

and non-smooth part term,

(15) $$g\left(W\right)=\beta {\left\|W\right\|}_{2,1}+\mu {\sum_{m=1}^{M}\left\|\sum_{t=1}^{T_{t}-1}\left(w_{t+1}-w_{t}\right)\right\|}_{1}$$

Then, the following function was used to approximate h(W)+g(W):

(16) $$\Omega \left(W,W_{i}\right)=h\left(W_{i}\right)+<W-W_{i},▽h\left(W_{i}\right)>+\frac{l}{2}{\left\|W-W_{i}\right\|}_{F}^{2}+g\left(W\right)$$

where ${\left\|\cdot \right\|}_{F}$ represents the F-norm of the matrix, $▽h(W_{i})$ denotes the gradient value at W during the i-th iteration, and l is the step size for each iteration. Finally, the APG algorithm can be updated using the following equation:

(17) $$W_{i+1}=\underset{W}{argmin}\frac{1}{2}{\left\|W-V_{i}\right\|}_{F}^{2}+\frac{1}{l}g\left(W\right)$$

(18) $$V_{i}=W_{i}-\frac{1}{l}▽h\left(W_{i}\right)$$

The value of l can be obtained through a linear search.

Based on previous experience, the process of using gradient descent to solve for W is often inefficient and prone to becoming stuck in local optima. Therefore, this paper adopts a method used in reference [24]. The search points used in this study were as follows:

(19) $$S_{i}=W_{i}+\Delta_{i}\left(W-W_{i-1}\right)$$

where $\Delta_{j}=\frac{p_{i-1}-1}{p_{i}},p_{i}=\frac{1+\sqrt[{2}]{1+4p_{i-1}^{2}}}{2}$.

### 2.4. Multi-Kernel Support Vector Machine

The SVM [26] seeks an optimal trade-off between model complexity and learning ability, making it effective in recognizing small sample sizes, high dimensions, and nonlinear models with good generalization capabilities. In this paper, we employed SVM to integrate features from different modalities and performed the final classification. First, for each modality of every participant, based on the selected brain regions, we can employ a multi-task feature selection method using longitudinal imaging and hypergraph to extract corresponding longitudinal features. Then, to facilitate subsequent classification and regression tasks, we concatenate the features from different time points together for each modality of each participant, resulting in a final feature vector: $\left\{{(X}_{i}^{\left(1\right)},\cdots ,X_{i}^{\left(m\right)},\cdots X_{i}^{\left(M\right)}),\;i=1,\cdots N\right\}$. The main idea of multi-kernel support vector machines is to construct a separate kernel for each type of data form. In this article, a corresponding kernel matrix was generated for each modal dataset:

(20) $$k_{m}(x_{m}^{i},x_{m}^{j})=\Phi_{m}(x_{m}^{i}{)}^{T}\Phi \left(x_{m}^{j}\right)$$

Then, by learning a mixed kernel on the basis of all individual linear combinations, we can obtain the fusion of multiple modalities’ kernel functions:

(21) $$k(x^{i},x^{j})=\sum_{m}^{M}\beta_{m}k_{m}(x_{m}^{i},x_{m}^{j})$$

Here, we adopt a linear kernel function to combine multiple kernel matrices into one mixed kernel matrix using $\beta_{m}$, where $\beta_{m}$ is the weighting coefficient, $\beta_{m}$ ≥ 0, and $\sum_{m}^{M}\beta_{m}=1$. In this article, we employed the grid search algorithm and performed tenfold cross-validation on the training set to find the optimal value for $\beta_{m}$. Finally, the SVM algorithm was employed for the ultimate classification. In this article, we utilized the LIBSVM toolkit [27] to solve the SVM classifier.

## 3. Experimental Procedure and Result Analysis

### 3.1. Experimental Setup

To validate the effectiveness of our THM2TFS method, we conducted three sets of experiments: AD vs. NC, MCI vs. NC, and AD vs. MCI. For each group of classification experiments, tenfold cross-validation was used to eliminate bias caused by random partitioning of the dataset. The LIBSVM library is employed to implement SVM classification, with all SVM parameters set to their default values. This article evaluates the classification results using four specific metrics: accuracy, sensitivity, specificity, and F1 score.

In addition, this study used the area under the Receiver Operating Characteristic (ROC) curve (AUC) as an evaluation metric. The AUC is a measure of performance for binary classification models and is commonly used to compare the performance of different models. The ROC curve shows the false positive rate (FPR) on the horizontal axis and the true positive rate (TPR) on the vertical axis. Each point on the ROC curve represents the values of the TPR and FPR for a classifier at different thresholds. The area under the ROC curve ranged between 0 and 1. An AUC of 1 indicates perfect classification by the model, while an AUC of 0.5 suggests that the model cannot distinguish between positive and negative cases. Generally, the larger the value of AUC, the better the model performance. In practical applications, if the AUC value of a model is greater than 0.7, it can be considered to have some level of classification ability. The purpose of using the AUC in this article is primarily to evaluate the model’s generalization ability, ensuring that the proposed method in this paper can achieve satisfactory classification performance on different datasets.

### 3.2. Experimental Results

To evaluate the classification performance of our proposed method, we performed each group classification task using single time-point images and combined images from four-time points, including (1) fMRI and sMRI data at baseline (T1), (2) fMRI and sMRI data at the 6th month (T2), (3) fMRI and sMRI data at the 12th month (T3), and (4) fMRI and sMRI data at the 24th month (T4). This was performed to validate the effectiveness of longitudinal analysis in diagnosing diseases more accurately.

#### 3.2.1. AD vs. NC Classification Results

**Figure 3:**
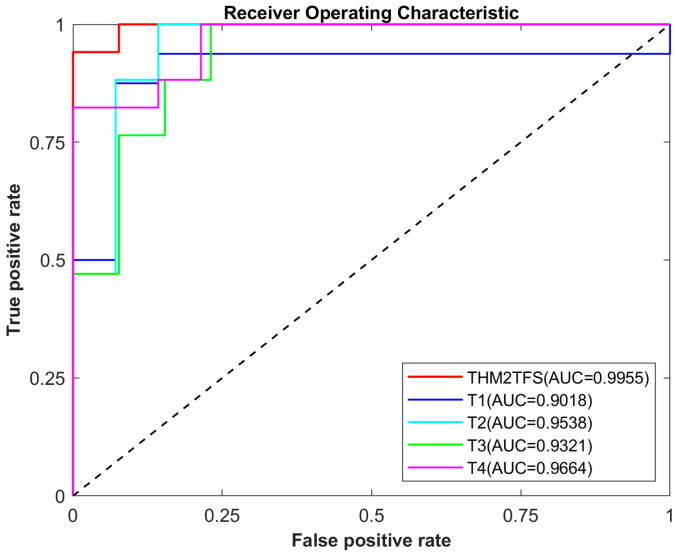
ROC curves of the AD vs. NC classification data at different time points.

Table 2 presents the results of the AD vs. NC classification tasks using single time-point images and four combined time-point images. The best results are highlighted in italics. As observed, performing classification tasks based on longitudinal imaging achieves optimal performance compared to a single time point. Specifically, the accuracy of THM2TFS (combining four time points of images) was 96.75%, with a sensitivity of 96.01%, specificity of 97.69%, F1 score of 97.12%, and AUC of 0.9955. Figure 3 illustrates the corresponding ROC curves for all individual time points and longitudinal imaging used for AD vs. NC classification. From this, it can be observed that our proposed method achieves optimal performance compared to single time points, with the highest AUC and highest true positive rate (TPR) along with a low false positive rate (FPR).

#### 3.2.2. AD vs. MCI Classification Results

**Figure 4:**
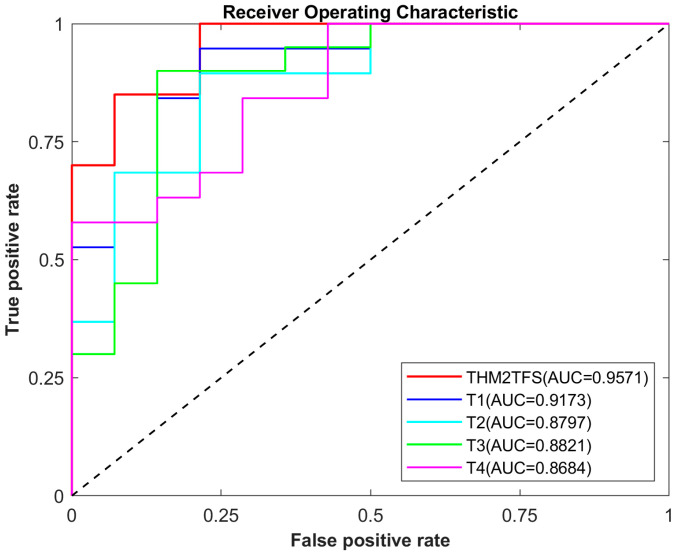
ROC curves of the AD vs. MCI classification data at different time points.

Table 3 presents the results of the AD and NC classification tasks using images with a single time point and images combined from four-time points. The proposed method achieved optimal performance in terms of accuracy, sensitivity, specificity, F1 score and AUC. We propose a method that achieves classification accuracies of 96.75% and 93.45% for the AD vs. NC classification task and AD vs. MCI classification task, respectively. This indicates that our proposed method can effectively distinguish between AD patients and NC or MCI patients. The ROC curves in Figure 4 also demonstrated that our method possesses certain advantages compared to other methods.

#### 3.2.3. MCI vs. NC Classification Results

**Figure 5:**
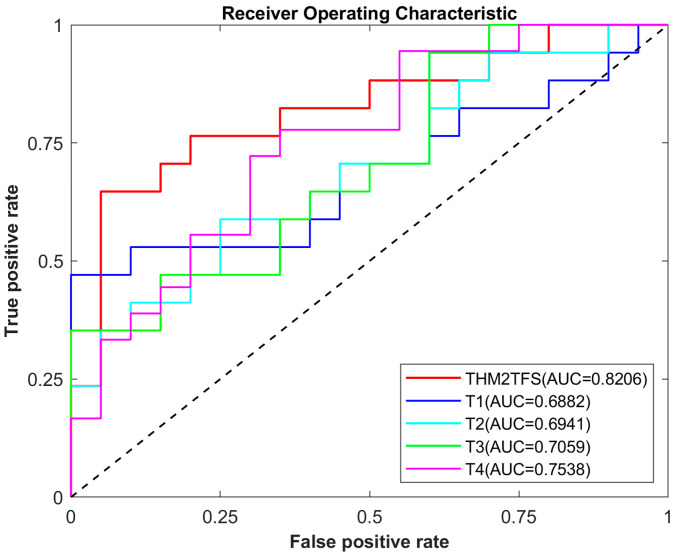
ROC curves of the MCI vs. NC classification data at different time points.

The classification results of MCI vs. NC are shown in Table 4. Our proposed method achieved the best classification performance compared to a single time point. The accuracy rate was 83.78%, the sensitivity was 77.78%, the specificity was 85%, the F1 score was 82.35% and the AUC was 0.8206. In addition, the results listed in Table 4 are generally lower than those in the AD vs. NC classification. This is because there are fewer changes in the brains of MCI patients than in those of AD patients. For example, MCI patients have much less hippocampal atrophy than AD patients. Therefore, MCI classification poses a more challenging task. The corresponding ROC curve is depicted in Figure 5, and it can also be observed that the classifier performance of our proposed method is superior.

### 3.3. Brain Region Analysis

**Figure 6:**
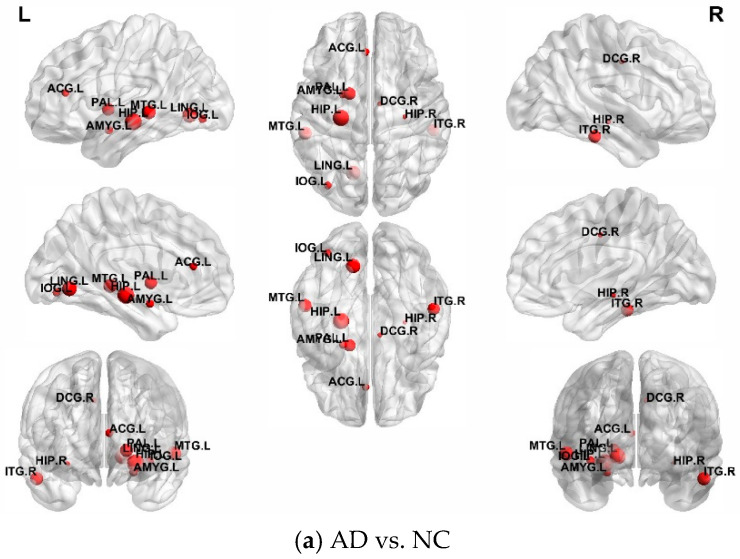

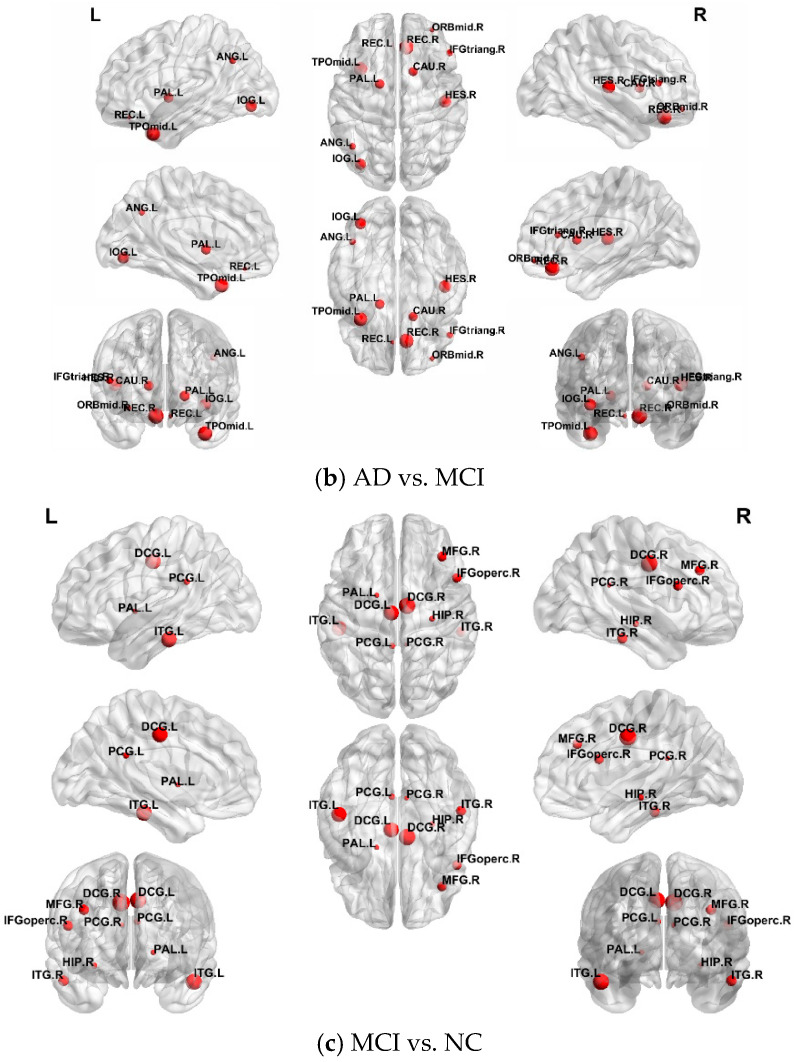
Discriminative brain regions in the different classification tasks.

The exploration of risk-related brain regions associated with AD is the focus of this study and also an important indicator for evaluating the superiority of algorithms [28,29]. The purpose of feature selection is to identify brain regions with high discriminative power. Since the selected brain regions vary in each cross-validation, this study separately counted the top 10 most frequently occurring brain regions in three classification experiments. Table 5 presents detailed information on the top 10 brain regions with the highest weights obtained by our proposed model. Table 6 shows the comparison table of full names and abbreviations of brain regions under the AAL template. The specific visualization effect is shown in Figure 6a–c. 

For the classification of AD and NC, our method selected the hippocampus, amygdala, temporal pole, and other brain regions. The hippocampus is a crucial structure within the brain that plays an important role in memory and spatial cognition. In patients with Alzheimer’s disease, hippocampal atrophy typically occurs early on, making it one of the pathological hallmarks of AD [30]. The amygdala is located in the temporal lobe’s temporal pole and is associated with emotions. Damage can lead to cognitive impairments in specific emotions [31]. The temporal pole has a wide range of functions, including auditory processing, memory, emotion and language, and is involved in several cognitive and perceptual processes [32].

For the classification of AD and MCI, the ROIs associated with AD included the temporal pole, paracentral lobule, and middle frontal gyrus. The paracentral lobule is associated with cognitive control, such as working memory, planning, and executing plans. Language plays a role in the formation and storage of both short-term and long-term memory, as well as in several cognitive and perceptual processes, particularly in the processing and memory of visual and linguistic information [33]. The middle frontal gyrus is a part of the cerebral cortex. It is an important region within the cerebral cortex that is associated with cognition, attention, and emotions [34].

In the classification tasks of MCI and NC, brain regions such as the hippocampus, pallidum, and cingulate gyrus were also selected. According to reports, the cingulate gyrus is involved in regulating human emotions and behaviors and is also associated with memory and cognitive functions [35]. The pallidum is a crucial brain region involved in controlling numerous vital neural functions, including motor control, reward systems, memory and learning, and emotional regulation.

Therefore, the brain regions selected by this method are indeed effective for diagnosing AD. The discovery of these regions can provide important information for the early diagnosis of AD and is expected to become a focal area of future research.

### 3.4. Effect of Different Regularization Terms on the Classification Performance

**Figure 7:**
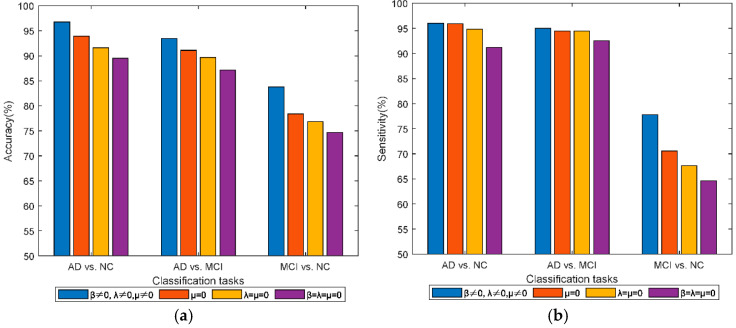

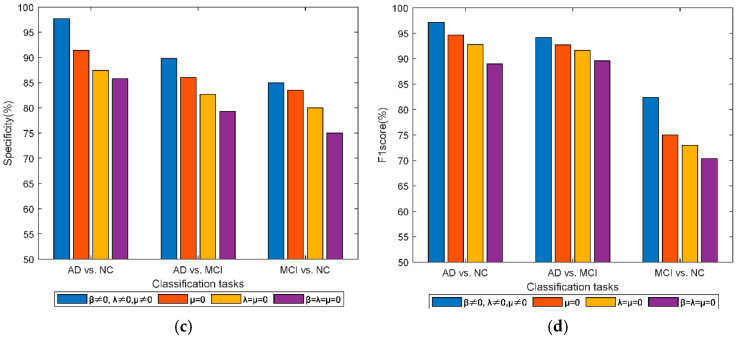
The changes in accuracy, sensitivity, specificity, and F1 score after introducing different regularization terms: (**a**) accuracy under different regularization conditions; (**b**) sensitivity under different regularization conditions; (**c**) specificity under different regularization conditions; and (**d**) F1 score under different regularization conditions.

In the objective function of our proposed THM2TFS method, there are three regularization terms, namely, the group sparsity regularization term β, hypergraph regularization term λ, and fusion smoothness regularization term μ. To investigate the effect of these three regularization terms on classification performance, we set one (or two or all three) of the regularization coefficients to zero and observe the changes in classification performance (accuracy, sensitivity, specificity, F1 score) for each of the three classification tasks. Figure 7, respectively, demonstrates the changes in accuracy, sensitivity, specificity, and F1 score after introducing different regularization terms. By introducing different regularization techniques, the accuracy, sensitivity, specificity, and F1 score of each group of classification tasks improved to varying degrees. Specifically, when β=λ=0, no regularization term is introduced; when λ=μ=0, only the group sparsity regularization term is introduced. Compared with not introducing any regularization term, the classification accuracy, sensitivity, specificity, and F1 score are all improved by introducing the group sparsity regularization term. This indicates that the group sparsity regularization term helps extract fewer relevant features and thus improves the accuracy of classification. When μ=0, that is, when both group sparse regularization and hypergraph regularization are introduced simultaneously, the model exhibits higher accuracy, sensitivity, specificity, and F1 score compared to previous cases. When β≠0, λ≠0, and μ≠0, that is, when all three regularization terms are introduced, the model’s classification accuracy, sensitivity, specificity, and F1 score are all superior to those of the previous three scenarios. This demonstrates the advantages of incorporating smoothness regularization.

### 3.5. The Effect of Different Hyperparameters on Classification Performance

In the proposed method, there are a total of four hyperparameters, including the number of nearest neighbors k and three regularization parameters (the group sparsity regularization term β, hypergraph regularization term λ, and fusion smoothness regularization term μ). The selection of hyperparameters is very important for model performance. When selecting hyperparameters, we refer to some existing studies, as well as the suitability of data structures and the tradeoff of computational complexity for reference, such as the *k*-nearest neighbor parameter used in creating hypergraphs, we choose to search in the range {3, 5, 7, 10, 15, 20, 25, 30} because, if the value of *k* is too small, the sample size of the super-edge connection is small, which may not fully reflect the complex relationship of the data. Too large a k value and too many samples connected by hyperedges will make the hypergraph structure complicated and may introduce noise, which makes it difficult to accurately reflect the real structure of data. And, if the value of k is too large, the computational complexity will also increase.

**Figure 8:**
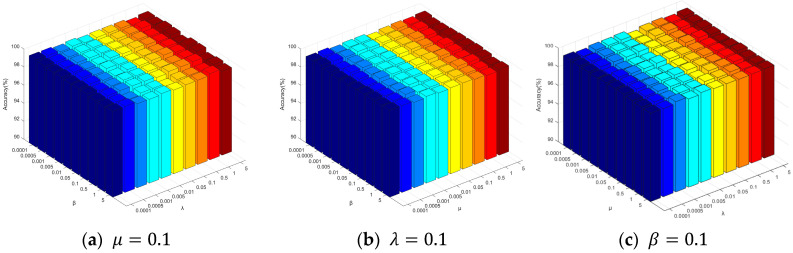
The effect of different parameters on the classification of AD and NC.

In this article, a grid search strategy is used to select the optimal parameter from the parameter set {0.0001, 0.0005, 0.001, 0.005, 0.01, 0.05, 0.1, 0.5, 1, 5}. To observe the sensitivity of specific parameters in the proposed method, a set of experiments (AD vs. NC) was conducted in this study to investigate the effect of these three regularization parameters on the classification results. First, one parameter was fixed at its optimal value, and, then the classification performance of the proposed method was compared under different values of the other two parameters. The experimental results are presented in Figure 8 (with k=7). From the experimental results in Figure 8, it can be observed that there is little fluctuation in the classification accuracy when parameters β, λ, and μ change. This indicates that the proposed method is relatively robust to regularization parameters and possesses a certain level of resilience.

**Figure 9:**
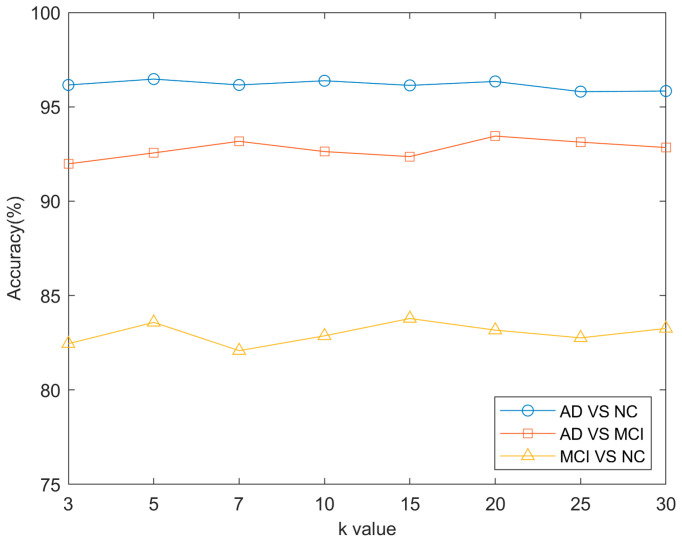
The effect of hyperedge size on the classification results between different classification tasks.

Although the choice of regularization parameter values is not particularly sensitive, the optimal β, λ, and μ parameters may vary slightly across different groups of classification tasks. Based on the optimal values of the β, λ, and μ parameters, we further analyzed the effect of the number of nearest neighbors (k) on the classification results for each group. The experimental results are shown in Figure 9, where the x-axis represents the number of nearest neighbors (k), ranging from {3, 5, 7, 10, 15, 20, 25, 30}, and the y-axis represents the classification accuracy. The different colored curves represent different classification tasks. From the graph, we can see that, as k increases, the curves for each color fluctuate insignificantly. This finding implies that the number of nearest neighbors (k) does not have a significant effect on the classification performance. Therefore, it can be concluded that the higher-order features extracted by hypergraph methods can effectively reflect the structure of the data.

### 3.6. Multimodal Classification

In recent years, researchers have discovered that different imaging modalities provide unique information from various perspectives, and these pieces of information complement each other. This approach enhances the accuracy of diagnosing brain diseases. Compared to single-modal methods, multimodal approaches often achieve better results. For further comparison with unimodal methods, this study conducted two additional sets of comparative experiments using only fMRI data and sMRI data for classification. The experimental results are shown in Table 7. From the table, it can be observed that, for all classification tasks, the classification performance using multimodal data is generally superior to that using only a single modality image. This is because multimodal image features achieve complementary information and provide more effective information.

### 3.7. Comparison with Existing Methods

We further compared the classification results obtained from our THM2TFS method with those obtained from studies combining multimodal feature selection and traditional machine learning for AD classification. It is worth noting that, for comparison, all studies used data from the ADNI dataset, with variations in the participants included in each study. Although the imaging data of the subjects used for research vary, the ADNI research team has implemented quality control and preprocessing on all image data. Therefore, our approach can be roughly compared with these existing methods to validate the effectiveness of the method that we propose. Table 8 shows that our proposed method outperforms most existing approaches in terms of AD and NC classification, AD and MCI classification, and MCI and NC classification. Therefore, the proposed multimodal feature selection method based on longitudinal data outperforms all single time-point data in terms of the results obtained from longitudinal data.

### 3.8. Model Complexity Analysis

In this study, we conduct a complexity analysis of the proposed model. The methodology in this paper consists of three key phases:

1. Multi-time point hypergraph construction: Assuming that each time point has n samples, d-dimensional features, and m modals, the time complexity of constructing hypergraphs is O($m\cdot n^{2}\cdot d$). Since the hypergraph is constructed independently at T = 4 time points, the total complexity is O($m\cdot T\cdot n^{2}\cdot d$) = O(4$m\cdot n^{2}\cdot d$).

2. Longitudinal feature selection: The objective function is optimized using near-end gradient descent, with each iteration containing the following operations: Gradient calculation: Complexity O($m\cdot d^{2}$), where m is the number of modes. Fused Lasso near-end operator: The longitudinal constraint on T = 4 time points requires an additional O(m·T·d) time to deal with the smoothness of adjacent time points. The total time complexity is O(T_iter_ ($m\cdot d^{2}$ + T·d)) = O(T_iter_ ($m\cdot d^{2}$ + 4d)), where T_iter_ is the number of iterations.

3. Feature fusion and classification: After longitudinal feature selection at four-time points of each mode, the dimension becomes D after feature concatenation, and, then, we undergo linear kernel SVM training with O($m\cdot n^{2}\cdot d$) complexity.

Therefore, the overall time complexity is O(4$m\cdot n^{2}\cdot d$) + O(T_iter_ ($m\cdot d^{2}$ + T·d)) + O($m\cdot n^{2}\cdot d$).

We make a simple comparison of the complexity of our model with existing methods, as shown in Table 9:

Despite the complexity of the proposed method due to multi-time-point hypergraphs and Fused Lasso, it is clinically necessary for the early diagnosis of AD to capture the dynamics of disease progression through longitudinal hypergraphs and to mine the time-dependent relationship of Fused Lasso.

## 4. Limitations and Future Work

In this study, we propose a multimodal feature selection algorithm based on longitudinal imaging and hypergraphs (THM2TFS) to jointly extract common features from longitudinal image data to improve the accuracy of AD classification. The method consists of four steps, namely, data preprocessing, hypergraph construction, multi-task feature selection, and multi-kernel classification. To validate our method, three sets of classification experiments were conducted using longitudinal sMRI and fMRI images from 512 subjects in the ADNI dataset at different time points ((baseline (T1), 6th month (T2), 12th month (T3), and 24th month (T4)). The results indicate that this method not only enables the joint extraction of common features in longitudinal data but also aids in identifying biomarkers associated with AD.

However, our study has limitations. First, the ADNI dataset contains demographic, neuropsychological, imaging, genetic, cerebrospinal fluid, and blood data collected from individual participants according to unified standards. In contrast, our method solely utilizes imaging data for classification. Then, we will develop techniques to handle incomplete data in terms of modality and time points, aiming to overcome the limitation of a small sample size for MCI patients and further enhance the final performance. Finally, we will only investigate the performance of binary classification problems and will not explore the performance of multivariate classification, which holds greater clinical significance. In multi-classification tasks, the existing multi-task feature selection and MKSVM methods are not suitable for multi-classification tasks and need to be improved. It can be extended to a multi-label multi-task learning model to process multiple categories of labels simultaneously. Deep neural networks are introduced to learn complex nonlinear relationships and enhance the multi-classification ability of the model. In the future, we will further enhance our research by integrating these three aspects and extend our methods to other applications such as diagnosing diseases such as cancer, Parkinson’s disease, and coronary heart disease.

## 5. Conclusions

In this study, we propose a multi-task feature selection algorithm for Alzheimer’s disease (AD) classification, based on longitudinal imaging and hypergraphs (THM2TFS). Our proposed method takes into account longitudinal data from multiple time points, which is different from most existing multi-task learning methods that focus on cross-sectional data analysis, such as using only single-time-point data. This method captures the intrinsic correlations between different tasks through multi-task learning, enabling the extraction of common features from longitudinal data. In particular, we introduce two regularization terms: (1) hypergraph Laplacian regularization, which preserves the high-order structural information of similar data to obtain more discriminative brain region features; and (2) Fused Lasso regularization, which adds temporal smoothness constraints between feature weights at adjacent time points to further reflect the longitudinal progressive changes in brain regions. Finally, we validated the performance of our proposed method on the ADNI dataset. As shown in the experimental results from Section 3.2, our method demonstrates better performance compared to learning methods that solely utilize single-time-point data. In addition, we listed the top 10 brain regions selected by our proposed method in Section 3.3. The results demonstrate that our approach can identify brain regions with high clinical relevance. Through the experiments in Section 3.4, we can demonstrate that the regularization term we introduced is more advantageous for feature selection. In Section 3.5, we investigated the effect of four different hyperparameters on classification performance, and the results indicate that the choice of parameter values is not particularly sensitive, thus proving that our proposed method possesses a certain level of robustness. Finally, in Section 3.6, we investigated the classification performance of multimodal data. Compared to single-modal data, multimodal data achieved better results, as demonstrated in Section 3.7 where our method showed superiority over several state-of-the-art AD classification methods. Our proposed approach can effectively classify AD/MCI cases.

## Figures and Tables

**Table 1:** Demographic data of the participants.

Subjects	AD	MCI	NC
Number	136	200	176
Gender (M/F)	80/56	132/68	90/86

**Table 2:** Comparison of results between AD and NC classifications at different time points.

Data	ACC	SEN	SPE	F1	AUC
T1	87.56	88.10	87.03	88.97	0.9018
T2	90.08	89.77	90.49	91.04	0.9538
T3	89.08	89.77	88.30	90.08	0.9321
T4	92.96	94.31	91.26	93.78	0.9664
**Proposed**	**96.75**	**96.01**	**97.69**	**97.12**	**0.9955**

**Table 3:** Comparison of results for AD and MCI classification at different time points.

Data	ACC	SEN	SPE	F1	AUC
T1	89.62	92.00	87.53	91.30	0.9173
T2	84.86	91.50	75.11	87.80	0.8797
T3	86.03	92.00	77.31	88.72	0.8821
T4	83.95	90.50	74.23	86.97	0.8684
**Proposed**	**93.45**	**95.00**	**89.78**	**94.15**	**0.9571**

**Table 4:** Comparison of results between MCI and NC classifications at different time points.

Data	ACC	SEN	SPE	F1	AUC
T1	72.33	62.97	80.50	67.57	0.6882
T2	73.72	63.14	83.00	69.32	0.6941
T3	75.26	67.12	82.50	71.59	0.7059
T4	76.88	67.65	85.00	72.85	0.7538
**Proposed**	**83.78**	**77.78**	**85.00**	**82.35**	**0.8206**

**Table 5:** The AAL atlas lists the top 10 brain regions that contribute the most to different classification tasks.

Sequence	AD vs. NC	AD vs. MCI	MCI vs. NC
1	**Hippocampus_L**	Rectus_R	**Cingulum_Mid_R**
2	Lingual_L	Temporal_Mid_L	**Cingulum_Mid_L**
3	Temporal_Mid_L	Heschl_R	Temporal_Inf_L
4	**Pallidum_L**	Occipital_Inf_L	Temporal_Inf_R
5	Temporal_Inf_R	Paracentral_Lobule_R	Frontal_Mid_R
6	**Amygdala_L**	Caudate_R	Frontal_Inf_Oper_R
7	Occipital_Inf_L	Angular_L	**Cingulum_Post_L**
8	**Cingulum_Ant_R**	Frontal_Inf_Tri_R	**Hippocampus_R**
9	**Cingulum_Mid_R**	Frontal_Mid_Orb_R	Pallidum_L
10	**Hippocampus_R**	Rectus_L	**Cingulum_Post_R**

**Table 6:** Comparison table of full names and short names of brain regions under the AAL template.

ROI Full Name	Micro-Number	ROIs Micro-Name
Right Middle Frontal Gyrus	8	Frontal_Mid_R
Right Inferior Frontal Gyrus, Opercular Part	12	Frontal_Inf_Oper_R
Right Inferior Frontal Gyrus, Triangular Part	14	Frontal_Inf_Tri_R
Right Superior Frontal Gyrus, Medial Orbital	26	Frontal_Mid_Orb_R
Left Gyrus Rectus	27	Rectus_L
Right Gyrus Rectus	28	Rectus_R
Right Anterior Cingulate and Paracingulate Gyri	32	Cingulum_Ant_R
Left Median Cingulate and Paracingulate Gyri	33	Cingulum_Mid_L
Right Median Cingulate and Paracingulate Gyri	34	Cingulum_Mid_R
Left Posterior Cingulate Gyrus	35	Cingulum_Post_L
Right Posterior Cingulate Gyrus	36	Cingulum_Post_R
Left Hippocampus	37	Hippocampus_L
Right Hippocampus	38	Hippocampus_R
Left Amygdala	41	Amygdala_L
Left Lingual Gyrus	47	Lingual_L
Left Inferior Occipital Gyrus	53	Occipital_Inf_L
Left Angular Gyrus	65	Angular_L
Right Paracentral Lobule	70	Paracentral_Lobule_R
Right Caudate Nucleus	72	Caudate_R
Left Lenticular Nucleus, Pallidum	75	Pallidum_L
Right Heschl Gyrus	80	Heschl_R
Left Middle Temporal Gyrus	85	Temporal_Mid_L
Left Inferior Temporal Gyrus	89	Temporal_Inf_L
Right Inferior Temporal Gyrus	90	Temporal_Inf_R

**Table 7:** Comparison of the classification results of different modalities.

Data	AD vs. NC				AD vs. MCI				MCI vs. NC			
	ACC	SEN	SPE	F1	ACC	SEN	SPE	F1	ACC	SEN	SPE	F1
fMRI	91.40	92.61	89.84	92.34	87.46	83.50	93.30	88.74	73.63	68.14	78.50	70.80
sMRI	92.99	90.98	95.66	93.48	88.14	87.00	89.84	89.57	75.25	65.33	84.00	70.52
fMRI + sMRI	**96.75**	**96.01**	**97.69**	**97.12**	**93.45**	**95.00**	**89.78**	**94.15**	**83.78**	**82.35**	**85.00**	**82.35**

**Table 8:** Comparison of classification accuracy with existing methods.

Method	Data	Classification Results (%)		
		AD vs. NC	AD vs. MCI	MCI vs. NC
Shao et al. [8]	MRI + FDG PET (single time)	92.51%	-	-
Huang et al. [36]	MRI + PET (single time)	94.30%	-	-
Lin et al. [37]	MRI + PET (single time)	89.26%	-	72.84%
Shi et al. [9]	MRI + PET (single time)	96.76%	-	80.73%
Ban et al. [10]	sMRI+FDG PET+AV-45 PET (single time)	**98.78%**	86.47%	78.15%
**Proposed**	**fMRI + sMRI (T1 + T2 + T3 + T4)**	96.75%	**93.45%**	**83.78%**

**Table 9:** Comparison of model complexity with existing methods.

Method	Complexity
M2TFS [38]	$O(T_{\mathrm{iter}}\;(m\cdot d^{2}))+O(n^{2}\cdot d$)
HM2TFS [8]	$O(m\cdot n^{2}\cdot d)+O(T_{\mathrm{iter}}\;(m\cdot d^{2}))+O(m\cdot n^{2}\cdot d$)
H*p*MTFS [10]	$O(m\cdot n^{2}\cdot d)+O(T_{\mathrm{iter}}\;(m\cdot d^{2}+p\cdot K\cdot d+d\cdot m))+O(m\cdot n^{2}\cdot d$)
**Proposed**	**O(4**$m\cdot n^{2}\cdot d$**) + O(T_iter_ (**$m\cdot d^{2}$ **+** 4d**)) + O(**$m\cdot n^{2}\cdot d$**)**

## Data Availability

Longitudinal sMRI and fMRI data from patients with Alzheimer’s disease and controls were downloaded from the Alzheimer’s Disease Neuroimaging Initiative (ADNI) database (http://adni.loni.usc.edu/ (accessed on 15 March 2022)).
